# Teen pregnancy in Inuit communities – gaps still needed to be filled

**DOI:** 10.3402/ijch.v75.31790

**Published:** 2016-12-09

**Authors:** Caroline Moisan, Chloé Baril, Gina Muckle, Richard E. Belanger

**Affiliations:** 1 Population Health and Optimal Health Practices Research Unit, CHU de Québec Research Centre, Université Laval, Ville de Québec, QC, Canada; 2 School of Psychology, Université Laval, Ville de Québec, QC, Canada; 3 Department of Pediatrics, Faculty of Medicine, Université Laval, Ville de Québec, QC, Canada

**Keywords:** Inuit, teen, adolescent, pregnancy, reproductive health, review, cross-cultural comparison

## Abstract

Teen pregnancy is depicted around the world as an important cause of health disparities both for the child and the mother. Accordingly, much effort has been invested in its prevention and led to its decline in the northern hemisphere since the mid-1990s. Despite that, high rates are still observed in the circumpolar regions. As Inuit communities have granted better understanding of teenage pregnancy a priority for the coming years, this article comprehensively reviews this multidimensional issue. By depicting current prevalence, likely determinants and possible impacts documented among Inuit of Canada, Alaska and Greenland, and contrasting them to common knowledge that has emerged from other populations over the years, great gaps surface. In some regions, the number of pregnancies per number of Inuit women aged between 15 and 19 years has increased since the turn of the millennium, while statistics from others are either absent or difficult to compare. Only few likely determinants of teenage pregnancy such as low education and some household factors have actually been recognized among Inuit populations. Documented impacts of early pregnancy on Inuit women and their children are also limited compared to those from other populations. As a way to better address early pregnancy in the circumpolar context, the defence for additional scientific efforts and the provision of culturally adapted sexual health prevention programmes appear critical.

Teenage pregnancy has been at the centre of extensive research. Every year, about 16 million children worldwide are born from mothers aged between 15 and 19 years. The World Health Organization describes teenage pregnancy as dangerous for the child as well as for the mother (1). In addition to physical and mental difficulties lived by both mother and child, namely, a higher rate of stillborn, neonatal deaths, postpartum haemorrhage, depression and anxiety, childbearing teenagers are often under the obligation to face decisions, such as abandoning school and, having a long-term impact on their personal life, their family and their community (2). For all these reasons, much effort from public health authorities is continually invested towards the prevention of these early pregnancies and the minimization of adverse reproductive outcomes (3).

In some circumpolar areas, the Inuit populations are young and are growing rapidly. For instance, the median age in Nunavut was 24 years in 2011, and 57% of the population from Nunavik was under 25 in 2014 (2,4). Despite promising perspectives, Inuit aged between 15 and 24 years are still at an increased risk for school abandonment, substance use and psychological distress (2). Inuit from Nunavik, as well as Inuit women from other Canadian regions, also generally have their first childbirth at an early age and have more children than other non-aboriginal women (5,6). Although the rate of teenage pregnancy has decreased in the northern hemisphere since the mid-1990s (7), high rates still observed in the circumpolar regions (8) establish teenage pregnancy as a major public health priority for Inuit communities (3).

This article aims to describe the current and available knowledge regarding teenage pregnancy among the Inuit populations of Canada, Alaska and Greenland. In describing the frequency of teenage pregnancy, likely determinants and possible impacts, and contrasting the situation of Inuit women with other populations, gaps in the literature, if present, will be highlighted. By taking account of the different cultural views on pregnancy among youth, we also believe that a broader understanding of the issue should emerge and pave the way for interventions and sexual health prevention programmes adapted to Inuit. This narrative review was conducted using the following databases: PubMed and Web of Sciences. The studies were selected based on the main inclusion criteria: assessing teenage pregnancy among Inuit or American Indian/Alaska Native (AI/AN). Reference lists of the included articles were also searched. To compare with other populations, governmental websites were included.

## Frequency

Teenage pregnancy encompasses both planned and unintended pregnancies and may terminate with live birth, foetal loss or induced abortion. Their frequency is usually reported as the number of pregnancies per number of women aged between 15 and 19 years. In circumpolar regions, their prevalence rates between Canadian, Alaskan and Greenlandic communities would have been expected to be equivalent at first, but differences were noted. As health authorities started to follow this issue in the 1970s (7), clear trends have emerged over the past 40 years underlining different social, cultural, geopolitical and public health contexts.

From 2003 to 2009, the rate of teenage pregnancy has grown from 118.8 to 161.3/1,000 in Nunavut, roughly four times higher than for other Canadian teens in the same year (7,9). In comparison to the Canadian population as a whole in 2009, 20% of births in Nunavut occurred among women aged between 15 and 19 years, in contrast with only 4% for Canadian women within the same age group (6). However, these territorial statistics, as with others depicted below, should not be attributed solely to the Inuit communities since they encompass both Inuit and non-Inuit women, and they are influenced by demography differences where the percentage of the population aged below 20 years old is higher in Inuit groups than in the general Canadian population.

For Nunavik, the most recent data depict a similar state. Between 2003 and 2007, an average of 83.5 pregnancies per 1,000 Inuit girls under 18 years compared to 14.2/1,000 for the whole Province of Quebec was reported in Duhaime's et al. (5) report. In the 18- to 19-year-old group, rates peaked at, respectively, 200.8/1,000 among Inuit communities and 57.5/1,000 for the whole provincial population. The highest Inuit pregnancy rate was observed among young adults aged between 20 and 24 years (237.6/1,000), while the highest pregnancy rate in Quebec was found in the 25–29 years age group (143.3/1,000), which is not directly related to teenage pregnancy, but depicts a high rate of childbearing at younger age among Inuit (5).

Very few reports regarding early pregnancy in Greenland are available. However, as exposed by Montgomery-Andersen et al. (10), after a decline in the 1990s, teen pregnancies have also increased in 2000, with a rate over 79 pregnancies per 1,000 girls aged between 15 and 19 years. Data extracted from Statbank Greenland (11) allow a more recent and concrete picture of pregnancy among girls aged 15–19 years having decreased from 150/1,000 live births in 2000 to 74/1,000 live births in 2015.

During the 1991–2010 period, birth rates significantly decreased, from 84.1 to 31.1 per 1,000 women, among AI/AN aged 15–19 years, which was lower than among non-Hispanic Black (39.0) and Hispanic (41.7) within the US population (12). Time trend statistics depicting specifically the situation among Inuit or among Alaskan Inuit only are essentially unavailable.

## Possible determinants

Among Inuit as well as other populations, low income and low education have been extensively studied and are common socio-economic factors associated with teenage pregnancy (13,14). Living in an overcrowded house and in a house in need of major repairs are the factors that have been linked to teenage pregnancy among Inuit and First Nations from Canada (15). As observed in Table I, several socio-economic factors related to teenage pregnancy in the general population have not yet been studied among Inuit, as well as among AI/AN.

**Table I:** Likely determinants known to be related to teenage pregnancies.

	Inuit	AI/AN	General population
Psychological and socio-economic factors			
Low education of teen	x	x	x
Single/divorced/separated/unmarried teen mother	x	x	x
Low household income	x	x	x
Single-parent household or large family living in teen's house	x	x	x
Overcrowding and house in need of major repairs	x		
Low education of teens’ parents			x
Divorced/separated parents of teen			x
Teenage pregnancy of the mother/siblings of the teen			x
Substance use of teen			x
Mental illness (distress, depression) of teen		x	x
Low self-esteem of teen			x
Sexual behaviours			
Lacking access to or making poor use of contraception		x	x
Early sexual activity		x	x
Frequency of sexual intercourse		x	x
Substance use before sexual intercourse		x	x
Diverse sexual risky behaviours (ever having sex, multiple partners, recent sexual intercourse)		x	x

AI/AN, American Indian/Alaska Native.

Specific sexual behaviours also predict higher risk of early pregnancy. Studies have depicted young AI/AN as being more likely to engage in risky sexual behaviours than the youth from the general urban population (16). AI/AN female youth do not have a higher prevalence rate of substance use during or before sexual intercourse than the general population (16), but they were more likely to have multiple sex partners, sexual intercourse before 13 years as well as being victim of forced sexual intercourse more frequently than White female youth (16,17). In an urban AI/AN group of women aged 15–24, 61% reported not currently using a contraceptive method (18). The Nunavik Inuit Health Survey reported that in 2004, 48% of 15- to 29-year-old Inuit women declared having used condoms at their last sexual intercourse (19). Cole (20), however, reported data showing that condom use rates were not different in young Inuit from Nunavut than among the general population. Unfortunately, there is no available literature only about Alaskan Natives regarding teen pregnancy.

Within literature examining non-Aboriginal populations, lacking access to or making poor use of contraception, substance use before sexual intercourse, sexual behaviours in exchange for some compensation and a high frequency of sexual activity are known risky sexual behaviours for teenage pregnancy among youth (21,22). Many family-related factors such as growing up in a single-parent household or a large family, low parental education, single-parent (divorced or separated) families and teenage pregnancy of the mother or siblings have also been associated with teenage pregnancy. Documented psychosocial factors linked to teenage pregnancy are aspects of self-esteem, attitude towards peers as well as tobacco, drug and alcohol use (23). Significant distress among female youth has also been related to an increasing risk of having unprotected sex and of being pregnant (24). Girls aged between 15 and 19 years diagnosed with a mental illness, such as depression, bipolar disorder and schizophrenia, are almost three times more likely to become pregnant as adolescents than those without such a diagnosis (25). There is no literature available assessing these risk factors for teenage pregnancy among Inuit.

Among Aboriginal youth, psychological factors such as self-efficacy to abstain from sex have been associated with delaying the onset of sexual activity. Cultural and social factors such as valuing school achievement, support from parents, having a positive Native identity and a sense of belonging to a Native community might also be protective against early pregnancy (26).

## Impact

As previously said, multiple risks, including mental and physical both for the mother and the child, are associated with early pregnancies (27,28). Among the chief medical problems is the fact that adolescent mothers are at extremely high risk for repeated pregnancy while still in adolescence (22). Although younger pregnant adolescents face more pregnancy complications, they are less likely to resort to prenatal care than older adolescents for various reasons, including denial of a possible pregnancy, lack of knowledge about the health care system or normal menstrual cyclicity, fear of the pregnancy itself or of informing their parents (22). Likewise, AI/AN teen mothers are less likely to turn to timely prenatal care, thus increasing the risk for premature birth and low birthweight (29). Past analyses have shown that their birth outcomes, such as low birth weight and infant mortality, were higher than that among White female youth, whether in the rural or urban population (30).

Inuit women are also at greater risk for preterm delivery (before 37 weeks gestation) and their rate of very-preterm delivery (before 32 weeks gestation) is much higher than the Canadian national average. More recent data, which might be only partly relevant to teen pregnancy, report preterm birth rates to be 1.7–1.8 higher than non-Aboriginal births. Inuit babies are at a higher risk for large gestational weight at birth and high birthweight, when compared to non-Aboriginal births, which are risk factors for perinatal death, infant mortality and postneonatal death (31). However, maternal characteristics only partially explain these neonatal health disparities as other plausible predictors such as environmental factors and lack of access to or use of social services may likely be implicated (32).

Numerous studies have shown that children of teenage mothers tend to have less favourable health outcomes than those of older mothers (33). Some of the problems might partially be related to lower parenting skills, poorer family support and lower income, while others are directly related to prematurity and its ensuing health complications and developmental problems. Unfortunately, few studies have addressed children's health problem taking into account the issue of the early age of Inuit mothers. Guèvremont (13), in her analysis of the population-based 2006 Aboriginal Children's Survey from Canada including Métis, Inuit and First Nations, found that 2- to 5-year-old Inuit children born from mothers having delivered their baby before the age of 20 compared to ≥25 years presented no differences in the incidence of chronic health conditions. While children from younger mothers were less likely to be found in excellent/very good health in this same study, this association disappeared after accounting for socio-economic factors. Nevertheless, children of Inuit teenage mothers were more likely than Inuit children born from older women to have had ear infections and dental problems. Likewise, prosocial behaviour as well as conduct problems and emotional distress during childhood does not appear to differ between Inuit children born from teenage mothers and those born from older mothers after accounting for socio-economic factors. Yet, the incidence of inattention-hyperactivity symptoms appears to be more severe among children born from younger Inuit mothers (13).

It has long been known that teenage mothers tend to be less educated and, thus, less employed, resulting in increased and longer dependence on welfare and public assistance (34). Inuit teenage mothers are more likely to have abandoned high school and to be single-mothers (13,32). The absence of a young father in the life of Inuit teenage mothers has in fact been frequently mentioned as a compounding problem of teenage pregnancies (8), adding to the already important burden of taking care of a young child. As a result, adult mothers who had their first child while still teenagers had a lower family income compared to Inuit women who had begun childbearing at an older age (15).

## Cultural insights

Teenage pregnancy has generally been accepted positively among Inuit communities and has been reported as being a part of traditional culture (35). Pregnancy and childbearing can be celebrated and found to enrich one's feminine role and may be seen as normal events of life, notwithstanding the mother's age in certain cultures (36,37). In that context, abandoning school to have a child appears to be more acceptable among Inuit than non-Inuit (35). According to this view, some teenage girls might deliberately decide to get pregnant or have no objection to becoming pregnant at an early age. Among Inuit in Nunavik, unplanned pregnancy and results of carelessness of becoming pregnant were common explanations of the high rate of teenage pregnancy obtained by Archibald (8). Some studies in diverse ethnic groups explain the occurrence of early pregnancy as a means to achieve adulthood, to find a purpose in life, to meet emotional needs, to keep away from drugs and alcohol, and to make healthy choices (8,37,38).

Adoption according to the traditional Inuit custom has strengthened the heart of the Inuit communities by building a large and interconnected family (39). In line, Inuit youth are more likely to view giving their children for adoption as an acceptable option for their pregnancy, compared to non-Inuit youth (20). Some community members and researchers have proposed that adoption nowadays might become a means to deal with teenage pregnancy (35,40). It has been demonstrated among Nunavik Inuit that adopted children growing up in families are less likely to experience maternal depression, alcohol abuse and domestic violence compared to non-adopted children (41). For some, adoption might be currently one of the best solutions to prevent mental health issues related to teenage pregnancy and minimize the social impacts of the latter in the northern regions (35).

Notwithstanding the means to deal with teen pregnancy, the number of abortions is high among northern communities. The abortion rate among teenage girls in Greenland is among the highest in the world, with 217 abortions/1,000 girls aged between 12 and 19 years in 2012 (42). The social and cultural norms, moral standards among communities and societies, as well as accessibility to abortion have been reported to influence the differences in abortion rate observed between the various northern regions (43).

There is a high prevalence of household overcrowding in Arctic countries, especially in Canada (44). Often, tenants on the waitlist for social housing are ranked according to “vulnerability” criteria such as living in an overcrowded house, having a low income and having dependent minor children (45). Anecdotal evidence suggests that it may be possible that social housing policies in the circumpolar North have, as an unintended consequence, an influence on early pregnancies, as having children might be a way for young women to access housing, to become independent and to move away from dysfunctional household (46). The impact of the “dependent minor children” criteria to access social housing as a way to improve one's living conditions on the attitudes towards early pregnancy is still unknown and could be the focus of more investigation.

In Canada and Alaska, the generation that lived through the resettlement phase and first lost contact with their Elders suffered from a lack of knowledge transfer, specifically regarding sexuality. The youngest generation of adults has suffered from this cultural break and had to learn about sexuality almost solely from medical resources, school system and internet (9,20,47). Inuit parents from Nunavut admitted having trouble explaining the concepts of a healthy sexual life to their teenaged children partly due to the traumatic experiences of residential schools (9). In 2004, the majority of Inuit students felt that they were not taught enough in school about sexually transmitted infections and contraception (20).

## Perspectives and implications

For an issue with such important public health implications, and since Inuit communities have shown a preoccupying prevalence of teen pregnancy over the past 40 years, a considerable amount of research effort remains to be invested. Yet, the poor monitoring of some populations, the lack of a clear understanding of the specific health determinants of Inuit youth and the failure to narrate inspiring stories on how communities have capitalized on effective individual and public interventions could be rapidly corrected.

There is a persisting problem in collecting evidence that would truly represent solid trends in pregnancy, abortion and live birth rates among Inuit population in Canada, Greenland and Alaska since the numbers hitherto presented are all but statistically comparable. Even though they have the highest rate of abortion (42), Greenland has larger gaps to fill in the literature, making it difficult to comprehensively portray teenage pregnancy in this region. Short-term outcomes of teenage pregnancy in the circumpolar context also remain imprecise both for mothers and their children. However, the “surveillance-type data” might not be the best to assess teenage pregnancy. Using other types of research methods, such as qualitative methods, may help obtaining social and culturally specific information to better address this issue (48). Socio-economic status issues, health services provided to communities, as well as local and national perspectives and policies regarding early pregnancy are only initial steps towards the understanding of the important differences in Inuit teen pregnancy rates between Greenland, Canada and Alaska.

The most effective initiatives in reducing rates of teen pregnancy in non-Aboriginal populations include (a) long-term community-based organizations to ensure sustainability and mobilization, (b) a focus on sexual education, sexual communication, ambivalence towards childbearing and delaying the onset of sexual activity and improving contraceptive accessibility, (c) appropriate training and (d) the involvement of stakeholders, youth and community partners (22,49). Results presented by McMahon et al. (50) with American Indian youth suggest six key components of an effective prevention initiative. First and second components: target the population (e.g. age and sex) and define the right timing. Third and fourth: determine the best location for implementation and involve trustworthy and role-model staff. Fifth and sixth: offer appropriate incentives and develop culturally appropriated content/activities using strengths-based approach. In direct connection with the latter proposition, the promotion of culturally adapted sexual health programmes has been on the rise in northern communities (8,48,51), and next health surveys should provide information to document their impact on rates of teenage pregnancies, sexually transmitted infections and contraception.

If contraceptive accessibility represents one of the main targets for more effective initiatives, financial planning to access all forms of effective contraceptive measures is already the standard in circumpolar regions, since all contraceptive methods are covered by the healthcare system. However, contraceptive accessibility, the assessment of adherence to contraceptive use and the prioritization of long-active reversible contraceptives should be irrespective of cultural or ethnic backgrounds. There is little to no literature on male involvement in sexual decision-making, such as the use of birth control, and it is much needed in Arctic communities.

Although Inuit have undergone major and stressful changes over the past 50 years, they are known for their tenacity and adaptability to changes (52). In the context of early pregnancy, the role of the community is especially important. Support from the community and from the circle of family and friends is precious not only to help teenage mothers to make healthier choices, but also to assist young mothers in having a good pregnancy experience or to mitigate its potential adverse impacts (8). To strengthen this social network, there is a need for more support from the educational and health systems as well as increased involvement of the elders in sexual education of teenage women (9).
